# The Vi Capsular Polysaccharide Enables *Salmonella enterica* Serovar Typhi to Evade Microbe-Guided Neutrophil Chemotaxis

**DOI:** 10.1371/journal.ppat.1004306

**Published:** 2014-08-07

**Authors:** Tamding Wangdi, Cheng-Yuk Lee, Alanna M. Spees, Chenzhou Yu, Dawn D. Kingsbury, Sebastian E. Winter, Christine J. Hastey, R. Paul Wilson, Volkmar Heinrich, Andreas J. Bäumler

**Affiliations:** 1 Department of Medical Microbiology and Immunology, School of Medicine, University of California, Davis, Davis, California, United States of America; 2 Department of Biomedical Engineering, University of California, Davis, Davis, California, United States of America; University of Michigan Medical School, United States of America

## Abstract

*Salmonella enterica* serovar Typhi (*S.* Typhi) causes typhoid fever, a disseminated infection, while the closely related pathogen *S. enterica* serovar Typhimurium (*S.* Typhimurium) is associated with a localized gastroenteritis in humans. Here we investigated whether both pathogens differ in the chemotactic response they induce in neutrophils using a single-cell experimental approach. Surprisingly, neutrophils extended chemotactic pseudopodia toward *Escherichia coli* and *S.* Typhimurium, but not toward *S.* Typhi. Bacterial-guided chemotaxis was dependent on the presence of complement component 5a (C5a) and C5a receptor (C5aR). Deletion of *S.* Typhi capsule biosynthesis genes markedly enhanced the chemotactic response of neutrophils *in vitro*. Furthermore, deletion of capsule biosynthesis genes heightened the association of *S.* Typhi with neutrophils *in vivo* through a C5aR-dependent mechanism. Collectively, these data suggest that expression of the virulence-associated (Vi) capsular polysaccharide of *S.* Typhi obstructs bacterial-guided neutrophil chemotaxis.

## Author Summary

To control a bacterial infection, neutrophils have to first migrate toward the microbe and then ingest and kill the intruder. Since *S.* Typhi has a greater propensity than *S.* Typhimurium to disseminate from its port of entry, we investigated whether both pathogens differ in their ability to evade neutrophil chemotaxis. Remarkably, *S.* Typhi, but not *S.* Typhimurium, inhibited neutrophil chemotaxis. Elucidation of the underlying mechanism revealed that microbe-guided chemotaxis proceeded through a C5a-dependent mechanism, which could be blocked by the Vi capsular polysaccharide of *S.* Typhi. These data suggest that the chemotactic chase of neutrophils is a host defense mechanism operational during gastroenteritis, but not during the initial stages of typhoid fever.

## Introduction

*Salmonella enterica* serovar Typhi (*S.* Typhi) is a strictly human-adapted pathogen associated with a disseminated febrile illness, termed typhoid fever (reviewed in [1]). In contrast, *S. enterica* serovar Typhimurium causes an infection that manifests as a localized gastroenteritis in immunocompetent individuals (Reviewed in [2]). Several *S.* Typhi virulence mechanisms that are absent from *S.* Typhimurium have been implicated to explain the differences in the disease presentation, including expression of the typhoid toxin [3], [4], altered flagellin gene regulation [5]–[7], altered invasion gene regulation [8]–[10] and expression of the virulence-associated (Vi) capsular polysaccharide [11]–[17]. In addition, *S.* Typhimurium genes that are absent from *S.* Typhi can contribute to differences in the outcome of host microbe interaction. These include the *gtgE* gene, encoding a type III secreted effector protein [18] and the *fepE* gene, which encodes a regulator of O-antigen chain length [19].

One of the host factors important for limiting dissemination of *S.* Typhimurium in humans appears to be neutrophils. The idea that neutrophils help prevent dissemination is supported by the finding that neutropenic individuals have an increased risk of developing invasive bloodstream infections with non-typhoidal *Salmonella* serovars (NTS) [20], [21]. Furthermore, severe malarial hemolysis impairs resistance to *S.* Typhimurium infection by impairing the neutrophil oxidative burst in a mouse model [22], which might explain the increased susceptibility of patients with severe pediatric malaria to develop bloodstream infections with NTS, most commonly *S.* Typhimurium [23]. Thus, while neutrophils help to limit dissemination of *S.* Typhimurium into the bloodstream in immunocompetent individuals, *S.* Typhi breaches this barrier to cause typhoid fever, suggesting that both pathogens differ in their ability to evade neutrophil-dependent host defense mechanism.

To eliminate a threat, neutrophils must migrate toward the microbial intruder and perform phagocytosis. Opsonophagocytosis is initiated when bacterial surfaces activate complement through the alternative pathway (reviewed in [24]). The complement component 3 (C3) cleavage product C3b is generated continuously at a low level. In the presence of bacteria, an internal thioester group of C3b reacts with a hydroxyl group on the bacterial surface to form an ester bond [25], [26]. The covalently surface bound C3b binds factor B to form C3bB, which is cleaved to yield the C3 convertase C3bBb (Fig. S1). In turn, surface bound C3 convertase promotes accelerated cleavage of C3 into C3a and C3b, thereby promoting opsonization by amplifying C3b deposition through ester bond formation with hydroxyl groups present on the bacterial surface. Finally, fragmentation of C3b leads to formation of C3bi, which is bound by complement receptor (CR)3, a phagocytic receptor expressed on the surface of neutrophils (reviewed in [27]). While it is well established that *S.* Typhi can overcome opsonophagocytosis [12], [16], it is not known whether *S.* Typhi and *S.* Typhimurium differ in their ability to evade neutrophil chemotaxis. Here we used single-cell experiments [28], [29] to study the chemotactic response of neutrophils toward *S.* Typhi and *S.* Typhimurium.

## Results

### Chemotactic responses of human neutrophils are obstructed by *S.* Typhi, but not by *S.* Typhimurium or *E. coli* Nissle 1917

**Figure 1:**
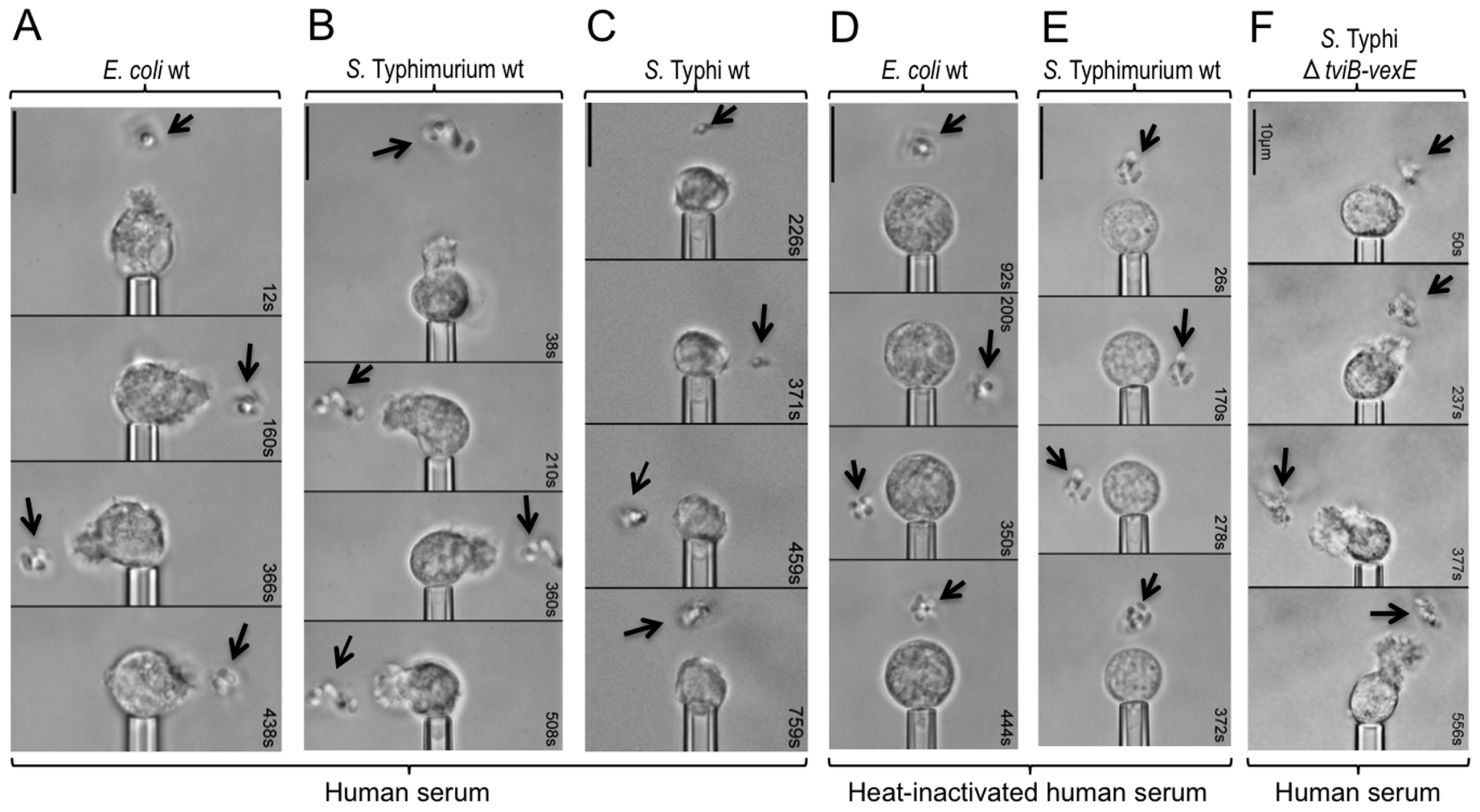
The Vi capsular polysaccharide inhibits chemotactic responses of human neutrophils *in vitro*. (A, B, C, D, E and F) The indicated bacterial strains were immobilized by laser tweezers (arrows) and brought in close proximity to a pipette-held human neutrophil. Video micrographs were taken at the indicated time points. At least four neutrophils were analyzed per blood sample. Each experiment was repeated with neutrophils from four different individuals and one representative example is shown. (A, B and F) Note chemotactic pseudopodia that extend from neutrophils toward bacteria.

To study bacterial-guided neutrophil chemotaxis, an initially quiescent human neutrophil was picked up at the tip of a micropipette. Bacteria were then immobilized by a laser optical-trap (laser tweezers) and brought stepwise into close proximity of the neutrophil in the presence of human serum (Fig. S2A). We investigated three organisms that interact differently with the human intestinal mucosa: a commensal organism residing in the intestinal lumen (*Escherichia coli* strain Nissle 1917), an invasive enteric pathogen associated with a localized gastroenteritis (*S.* Typhimurium) and the causative agent of an invasive disseminated infection termed typhoid fever (*S.* Typhi). Bringing *E. coli* (Fig. 1A, Video S1) or *S.* Typhimurium (Fig. 1B, S2B, Video S2) into a certain distance of a human neutrophil induced a vigorous chemotactic response, characterized by formation of a cellular pseudopod, which protruded toward the bacteria and responded quickly to their relocation. In striking contrast, *S.* Typhi did not elicit any chemotactic response by human neutrophils (Fig. 1C, S2C, Video S3).

### Bacterial-guided chemotactic responses of human neutrophils are complement-dependent

We reasoned that the chemotactic response toward *E. coli* or *S.* Typhimurium could be due to the release of *N*-formyl peptides from the bacterial surface, which are known neutrophil chemoattractants [30]. Alternatively, bacterial surface carbohydrates might activate complement by the alternative pathway, thereby giving rise to complement fragment C5a, a potent neutrophil chemoattractant [31]. Only the latter mechanism depends on the presence of serum proteins, the activity of which can be abrogated by heat inactivation of serum. Specifically, one protein in the alternative pathway of complement activation that is heat-sensitive is factor B (reviewed in [32]). As a result, heat inactivation of serum prevents the generation of C3 convertase (i.e. surface bound C3bBb), which promotes cleavage of C3 into C3a and C3b (Fig. S1). A downstream consequence of preventing formation of C3 convertase is that C3b can no longer bind surface bound C3bBb to form C5 convertase (i.e. surface bound C3bBbC3b), which promotes cleavage of C5 into C5a and C5b. Through this chain of events, heat inactivation of serum abrogates the formation of C5a. Interestingly, in the presence of heat-inactivated human serum, neither *E. coli* (Fig. 1D, Video S4) nor *S.* Typhimurium (Fig. 1E, Video S5) elicited a chemotactic response from human neutrophils in single-cell experiments.

**Figure 2:**
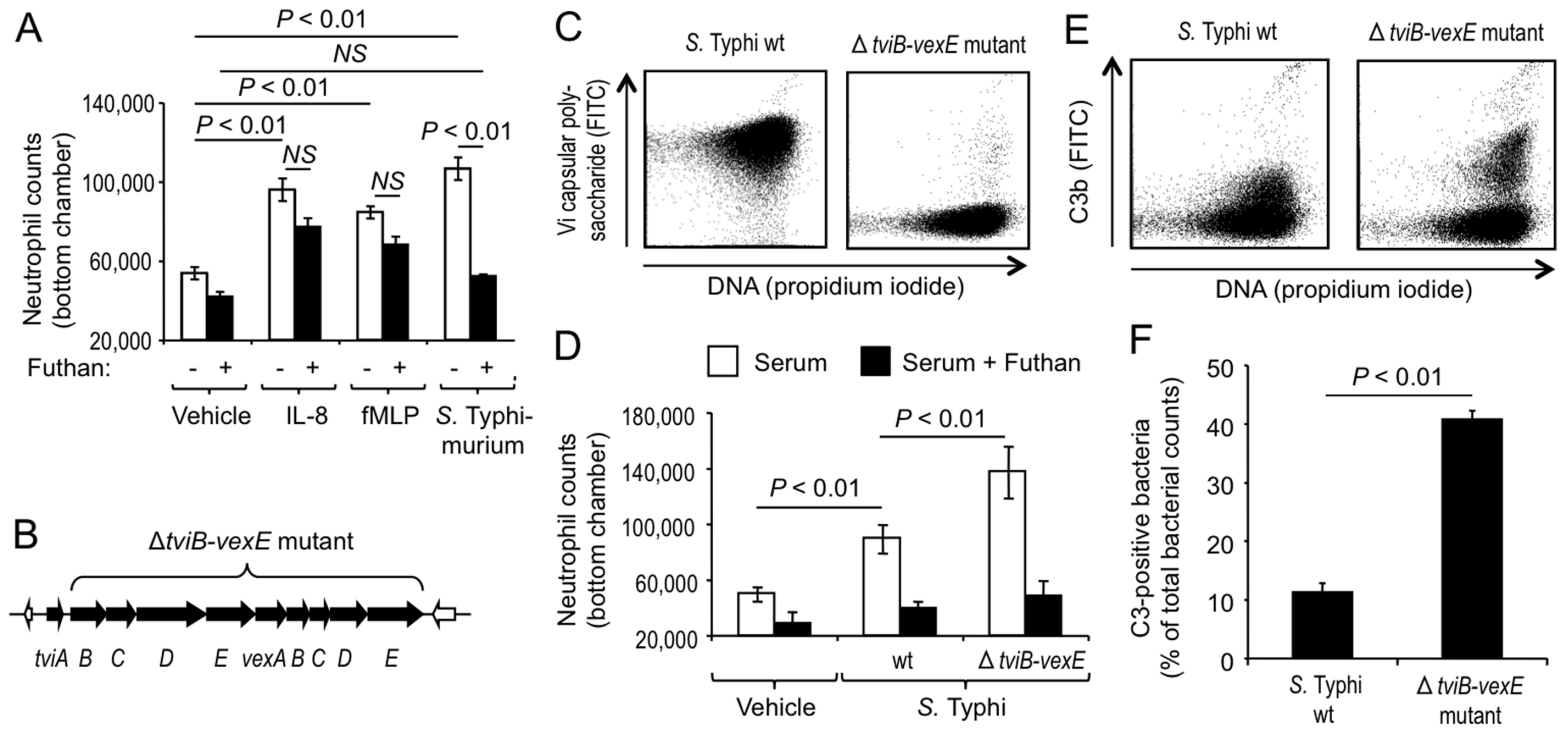
The Vi capsular polysaccharide inhibits complement-dependent chemotaxis *in vitro*. (A and D) Neutrophil migration into the bottom compartment of a Boyden chamber containing the indicated attractants was measured in the presence or absence of the complement inhibitor Futhan. Bars represent averages ± standard error from at least 3 different donors. Vehicle; HBSS vehicle control, fMLP; *N*-formyl-L-methionyl-L-leucyl-L-phenylalanine; wt, wild type. (B) Schematic drawing of Vi capsular biosynthesis genes (black arrows) of *S.* Typhi. (C) Expression of the Vi capsular polysaccharide (Y-axis) was detected in cultures of the *S.* Typhi wild type (wt, left panel) and a non-capsulated *S.* Typhi strain (Δ*tviB-vexE* mutant, right panel) by flow cytometry using rabbit anti-Vi serum. (E) Fixation of C3b (Y-axis) on the surface of the *S.* Typhi wild type (wt, left panel) or a non-capsulated *S.* Typhi strain (Δ*tviB-vexE* mutant, right panel) that had been incubated for 30 minutes in 1% human serum was detected by flow cytometry using FITC-conjugated goat anti-human C3b antibody. (F) Quantification of C3b fixation detected by flow cytometry. A gate was set using a control in which bacteria were not stained with goat anti-human C3b antibody. Bars represent averages ± standard error from three independent experiments for each strain.

To further investigate the mechanism of bacterial-guided neutrophil chemotaxis we used a standard Boyden chamber assay [33]. In this assay, the migration of human neutrophils from the upper compartment of the Boyden chamber [33] into a bottom reservoir that contains human serum is measured in the presence or absence of chemoattractants. The presence in the bottom chamber of either *S.* Typhimurium, IL-8 or synthetic *N*-formyl peptide (N-formyl-L-methionyl-L-leucyl-L-phenylalanine, fMLP) elicited migration of significantly (*P*<0.01) larger numbers of human neutrophils into the bottom reservoir than the vehicle control (Fig. 2A). To investigate the contribution of complement we used Futhan, which specifically binds factor Bb in C3 convertase (i.e. surface bound C3bBb) and C5 convertase (i.e. surface bound C3bBbC3b), thereby inhibiting the production of C3a and C5a (Fig. S1) [34], [35]. Addition of the complement inhibitor Futhan blunted the *S.* Typhimurium-induced neutrophil migration into the bottom chamber (*P*<0.01) (Fig. 2A). In contrast, migration of human neutrophils elicited by IL-8 or *N*-formyl peptide was not significantly inhibited by the addition of Futhan (*P*>0.05). Collectively, these data suggested that neutrophil chemotaxis elicited by *S.* Typhimurium was mediated by a complement-dependent mechanism, which was distinct from the complement-independent mechanisms of neutrophil chemotaxis elicited by IL-8 or *N*-formyl peptide.

### The Vi capsular polysaccharide of *S.* Typhi impairs complement-dependent chemotactic responses of neutrophils *in vitro*

We next wanted to investigate the mechanism by which *S.* Typhi obstructs neutrophil chemotaxis. One major difference to the closely related *S.* Typhimurium is that *S.* Typhi expresses the Vi capsular polysaccharide [36]. Expression of the Vi capsular polysaccharide in *S.* Typhi is encoded by the *viaB* locus on *Salmonella* Pathogenicity island 7 (SPI7), a DNA region that is absent from the *S.* Typhimurium genome [37]. The *viaB* locus contains genes for the regulation (*tviA*), the biosynthesis (*tviBCDE*) and the export (*vexABCDE*) of the Vi capsular polysaccharide (Fig. 2B) [38]. To investigate a possible role of the Vi capsular polysaccharide in evading neutrophil chemotaxis we used single-cell experiments with human neutrophils (Fig. S2A) to compare chemotactic responses elicited by the capsulated *S.* Typhi wild type (Ty2) with those elicited by an isogenic strain lacking capsule expression due to deletion of the capsule biosynthesis and export genes (Δ*tviB-vexE* mutant) (Fig. 2C) [5]. In striking contrast to the capsulated *S.* Typhi wild-type, which did not elicit neutrophil chemotaxis (Fig. 1C, S2C, Video S3), human neutrophils readily extended chemotactic pseudopodia towards the non-capsulated *S.* Typhi Δ*tviB-vexE* mutant (Fig. 1F, S2C, Video S6).

To further investigate whether expression of the Vi capsular polysaccharide inhibits neutrophil chemotaxis, we monitored migration of human neutrophils from the upper compartment of a Boyden chamber [33] into a bottom reservoir that contained human serum inoculated with vehicle control or different bacterial strains. The presence of the non-capsulated *S.* Typhi Δ*tviB-vexE* mutant in the bottom chamber elicited migration of significantly (*P*<0.05) larger numbers of human neutrophils than the presence of the capsulated *S.* Typhi wild-type strain (Fig. 2D). Furthermore, neutrophil migration towards bacteria was impaired in the presence of the complement inhibitor Futhan. Collectively, these data suggested that *S.* Typhi obstructs complement-dependent chemotactic responses of human neutrophils *in vitro* by expressing the Vi capsular polysaccharide. We next investigated whether the Vi-capsular polysaccharide also obstructs chemotactic responses of murine neutrophils. While the capsulated *S.* Typhi wild type did not elicit chemotactic responses from murine neutrophils (Fig. S3A, Video S7), murine neutrophils extended chemotactic pseudopodia towards the non-capsulated *S.* Typhi Δ*tviB-vexE* mutant (Fig. S3B, Video S8).

**Figure 3:**
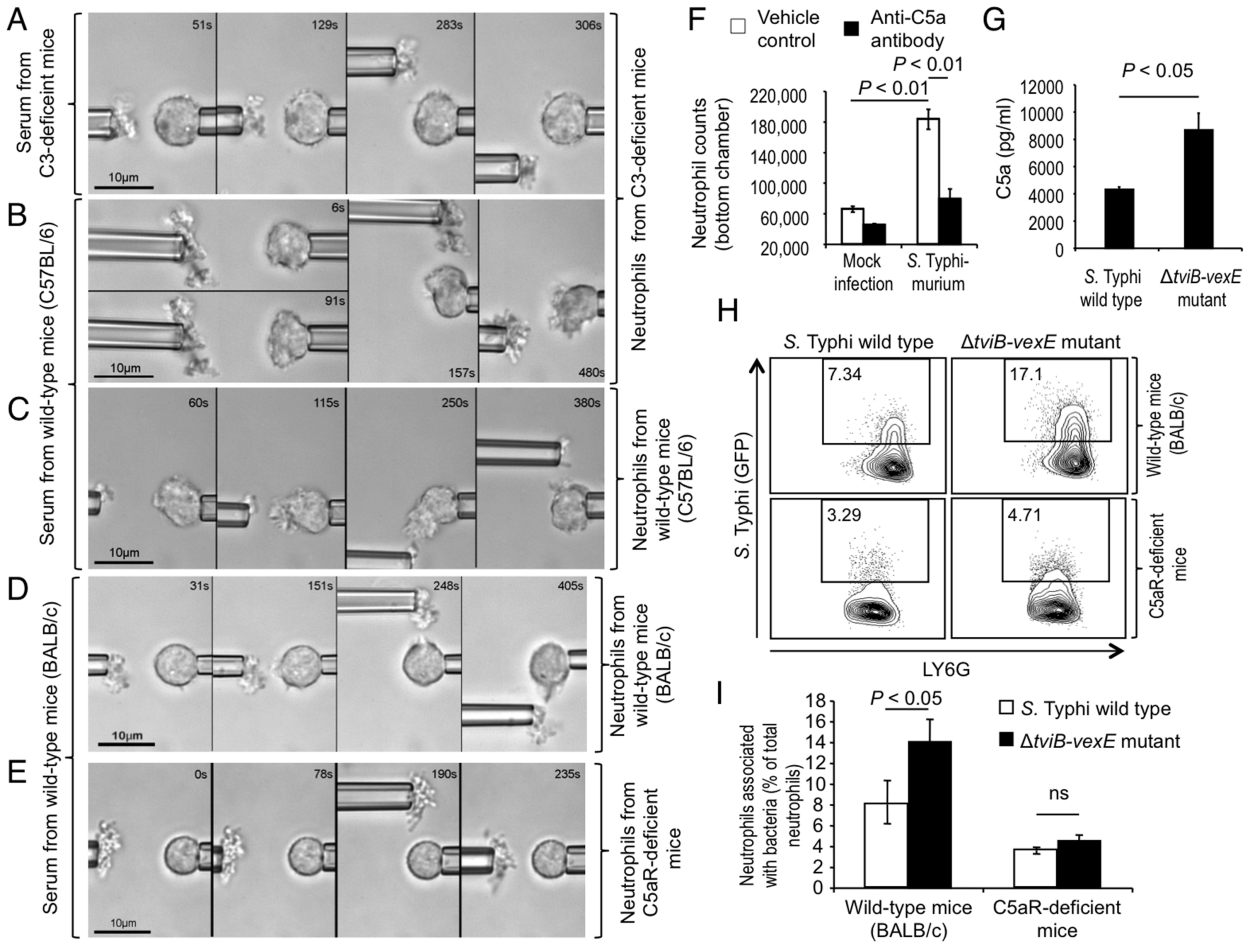
The Vi capsular polysaccharide inhibits chemotactic responses of murine neutrophils *in vitro* and *in vivo*. (A–E) Video micrographs of single-cell experiments with an agglutinated non-capsulated Δ*tviB-vexE* mutant and pipette-held murine neutrophils from the mouse strains indicated on the right. The assay was performed in buffer containing serum from the mouse strains indicated on the left. Blood from 4 animals was pooled for isolation of serum and neutrophils for an experiment. Each experiment was repeated at least four times and one representative example is shown. (F) Migration of human neutrophils into the bottom compartment of a Boyden chamber containing the indicated attractants was measured in the presence or absence of anti-C5a antibody. Bars represent averages ± standard error from three experiments. (G) Generation of C5a was detected by ELISA 15 minutes after incubation of the indicated *S.* Typhi strains in human serum. Bars represent averages ± standard error from experiments. (H and I) Mice were infected intraperitoneally with the indicated GFP-labeled bacterial strains and cells were collected one hour later by intraperitoneal lavage. Representative images of bacterial association (Y-axis) with neutrophils from wild type mice or congenic C5aR-deficient mice (H) and quantitative analysis of the data (averages ± standard error) from groups of six animals (I) are shown. ns, not significantly different.

Expression of the Vi capsular polysaccharide reduces complement activation by the alternative pathway [12], [16], as indicated by diminished fixation of complement fragment C3b on the surface of the capsulated *S.* Typhi wild-type strain compared to a non-capsulated *S.* Typhi mutant (Δ*tviB-vexE* mutant) (Fig. 2E and 2F). To further investigate whether chemotactic responses were dependent on C3, we performed single-cell experiments with serum and/or neutrophils from mice with C3-deficiency [39]. In serum from C3-deficient mice (bred on a C57BL/6 background), neutrophils from C3-deficient mice did not exhibit a chemotactic response toward the non-capsulated *S.* Typhi Δ*tviB-vexE* mutant (Fig. 3A, Video S9). In contrast, neutrophils from C3-deficient mice extended chemotactic pseudopodia toward the non-capsulated *S.* Typhi Δ*tviB-vexE* mutant when the experiment was performed in serum from wild type (C57BL/6) mice, which contains functional C3 protein (Fig. 3B, Video S10). As expected, chemotactic responses were also observed when both serum and neutrophils were derived from wild type (C57BL/6) mice (Fig. 3C, Video S11).

We next investigated chemotactic responses of neutrophils from C5aR-deficient mice (bred on a BALB/c background), which exhibit a specific defect in complement-dependent chemotaxis [40]. C5aR (also known as CD88) induces chemotactic responses by binding the serum component C5a, a soluble cleavage product of C5, which is generated during activation of the complement cascade (Fig. S1) [31]. Unlike neutrophils from wild-type mice (BALB/c) (Fig. 3D, Video S12), neutrophils from C5aR-deficient mice did not exhibit any chemotactic responses toward the non-capsulated *S.* Typhi Δ*tviB-vexE* mutant (Fig. 3E, Video S13). Collectively, these results showed that similar to what was observed with human neutrophils (Fig. 2D), chemotactic responses of murine neutrophils toward the non-capsulated *S.* Typhi Δ*tviB-vexE* mutant were complement-dependent.

To directly investigate the contribution of C5a to neutrophil chemotaxis, we monitored migration of human neutrophils from the upper compartment of a Boyden chamber into a bottom reservoir that either contained vehicle control or a neutralizing mouse anti-human C5a antibody. The presence of *S.* Typhimurium in the bottom chamber elicited neutrophil migration, which was significantly (*P*<0.01) reduced in the presence of anti-C5a antibody (Fig. 3F). In single cell experiments, anti-C5a antibody markedly blunted the chemotactic response of human neutrophils towards *S.* Typhimurium (Fig. S4). Furthermore, expression of the Vi capsular antigen reduced (*P*<0.05) generation of C5a during incubation of *S.* Typhi in human serum (Fig. 3G). Collectively, the outcome of these *in vitro* experiments suggested that the chemotactic responses of neutrophils towards bacteria were C5a-dependent.

### Neutrophil chemotaxis is required for bacterial uptake *in vivo*

Since *in vitro* experiments with murine neutrophils recapitulated our results obtained with human neutrophils, we proceeded to investigate whether the Vi capsular polysaccharide could inhibit neutrophil chemotaxis *in vivo* using a mouse model. *S.* Typhi is a human restricted pathogen and is rapidly cleared from organs of mice [16]. However, since chemotactic chasing by neutrophils is an early event that precedes clearance of bacteria, we reasoned that this aspect of host microbe interaction could be studied in mice at early time points after infection. The capsulated *S.* Typhi wild-type strain and the non-capsulated *S.* Typhi Δ*tviB-vexE* mutant were transformed with a plasmid encoding green fluorescence protein (GFP) and injected intraperitoneally into mice (BALB/c). Both GFP-labeled bacterial strains were recovered in similar numbers from the peritoneal cavity one hour after infection (Fig. S5A). Neutrophils present in the peritoneal lavage one hour after infection were analyzed by flow cytometry (Fig. S6). Both GFP-labeled bacterial strains elicited the influx of similar numbers of neutrophils into the peritoneal cavity (Fig. S5B and S5C). However, a significantly larger fraction of neutrophils were associated with the non-capsulated *S.* Typhi Δ*tviB-vexE* mutant than with the capsulated *S.* Typhi wild-type strain (Fig. 3H and 3I). These data suggested that expression of the Vi capsular polysaccharide impaired the ability of neutrophils to take up bacteria *in vivo*.

To determine whether these differences were due to a capsule-mediated inhibition of neutrophil chemotaxis, the experiment was repeated in C5aR-deficient mice, which are defective for complement-dependent chemotactic responses (Fig. 3E) [40]. When C5aR-deficient mice were infected with the capsulated *S.* Typhi wild-type strain or the non-capsulated *S.* Typhi Δ*tviB-vexE* mutant, there was a marked overall reduction in the fraction of neutrophils associated with GFP-labeled bacteria. Furthermore, no differences were observed in C5aR-deficient mice between the fractions of neutrophils associated with capsulated or non-capsulated bacterial strains (Fig. 3H and 3I), indicating that in the absence of complement-dependent chemotaxis, the Vi capsular polysaccharide had no detectable influence on bacterial association with neutrophils early after infection. Collectively, these data suggested neutrophils engulf bacteria in the peritoneal cavity through a C5Ra-dependent mechanism that can be obstructed by *S.* Typhi through expression of the Vi capsular polysaccharide.

## Discussion

Neutrophil migration into an infected tissue is initially guided by chemoattractants, such as interleukin (IL)-8, a chemokine produced by host cells after they detect the presence of bacteria using pathogen recognition receptors (PRRs). Neutrophils that follow a gradient of IL-8 to enter a site of infection will ultimately reach host cells producing this chemokine. In Boyden chamber assays, IL-8 can contribute to host cell migration, because this chemokine can be released from neutrophils upon contact with bacteria [41]. However, early microscopic observations suggest that chemotactic chasing of *Staphylococcus aureus* by neutrophils is bacterial-guided [42]. Investigation of bacterial factors possibly involved in this process suggests that purified C5a and *N*-formyl-l-methionyl-l-phenylalanine both can cause a change in neutrophil shape from a spherical to a polarized configuration [43], [44]. Some studies on the mechanism that triggers chemotaxis towards intact bacterial cells suggest a contribution of C5a [45], [46], while others suggest that a release of *N*-formyl peptides from the surface of intact bacterial cells is involved [41], [47]–[51]. Thus, it is not clear from the literature whether C5a emanating from the microbial surface, the release of *N*-formyl peptides from the bacterial cell or a combination of both mechanisms account for *Salmonella*-guided neutrophil chemotaxis. Here we addressed this question using microscopic observations of single cell experiments. Our results show that the formation of chemotactic pseudopodia towards intact cells of *S.* Typhimurium or non-capsulated *S.* Typhi was solely dependent on complement.

The question of whether either complement or *N*-formyl peptides are responsible for *Salmonella*-guided neutrophil chemotaxis is relevant because expression of the Vi capsular polysaccharide prevents complement deposition on the bacterial surface [12], [16], but is not known to inhibit detection of *N*-formyl peptides by neutrophils. The Vi capsular polysaccharide is a homopolymer composed of α-1,4 (2-deoxy)-2-*N*-acetyl-3-*O*-acetylgalacturonic acid, which is devoid of free hydroxyl-groups that would be available for C3b deposition (Fig. S1) [52]. In other words, by covering its surface with Vi capsular polysaccharide, *S.* Typhi inhibits activation of complement through the alternative pathway by preventing covalent binding of C3b to the bacterial cell. The *fepE* gene, which is required for expression of very long O-antigen chains containing an estimated 100 copies of a oligosaccharide repeat unit, is interrupted in the *S.* Typhi genome by a stop codon. Restoration of the *fepE* gene in *S.* Typhi results in expression of very-long O-antigen chains, which in turn results in enhanced C3 fixation [19], indicating that capsular material must extend beyond the O-antigen chains to effectively inhibit complement activation. Thus, inhibition of complement activation is not a property inherent to capsules, but might be a function of their length and carbohydrate composition. For example, the capsular polysaccharide of *Cryptococcus neoformans* prevents neither complement activation nor neutrophil chemotaxis [53]. In contrast, the Vi capsular polysaccharide of *S.* Typhi inhibits complement-dependent processes, such as opsonophagocytosis [12], [16]. Here we show that inhibition of complement activation by the Vi capsular polysaccharide also abrogated bacterial-guided neutrophil chemotaxis, because this process was solely complement dependent. Our supplementary videos illustrate that the Vi capsular polysaccharide can act as a “cloaking device” that makes *S.* Typhi practically “invisible” to neutrophils. By using an animal model we were able to show that C5aR-dependent chemotaxis is essential for a rapid association of bacteria with neutrophils. Obstruction of C5aR-dependent chemotaxis by the Vi capsular polysaccharide thus aids in evading neutrophil-dependent host defense mechanisms.

Our results suggest that one of the differences between gastroenteritis and typhoid fever is that the pathogen causing the latter disease evades neutrophil chemotaxis. A correlation between blocked neutrophil chemotaxis and increased vulnerability to bacterial dissemination is also observed during gonorrhea, where delayed stimulation of complement-dependent neutrophil chemotaxis *in vitro* is associated with dissemination of *Neisseria gonorrhoeae* infection [46]. Furthermore, C5aR mediates mucosal defense in a mouse model of *Pseudomonas aeruginosa* lung infection [40]. The inhibition of neutrophil chemotaxis reported here likely cooperates with other functions ascribed to the Vi capsular polysaccharide, including an obstruction of osponophagocytosis [12], [16] and a blunting of the respiratory burst of neutrophils [11], to help *S.* Typhi overcome neutrophil-dependent mucosal barrier functions. However, the increased ability of *S.* Typhi to cause bacteremia in immunocompetent individuals likely involves additional Vi capsular polysaccharide-independent mechanisms, such as changes in flagella gene expression, which have been implicated in increasing bacterial dissemination in animal models [6], [7].

## Materials and Methods

### Ethics statement

All animal experiments were performed according to USDA guidelines and approved by the Institutional Animal Care and Use Committee at the University of California at Davis. The University of California at Davis Institutional Review Board approved the protocol for obtaining blood draws for this study and written informed consent was obtained from all individuals.

### Bacterial strains and culture conditions

The *S.* Typhi wild-type isolate Ty2 (ATCC 19430) was obtained from the American Type Culture Collection. A derivative of Ty2 carrying a Δ*tviB-vexE* deletion (SW74) has been described previously [5]. To label bacteria with GFP, *S.* Typhi strains were transformed with plasmid pDW5 [54]. All bacterial strains were maintained in −80°C freezer stocks and were streaked on Luria-Bertani (LB) agar plates (15 g/l agar, 10 g/l tryptone, 5 g/l yeast extract, 10 g/l NaCl). To induce optimal expression of Vi capsular polysaccharide, strains were grown overnight in modified LB broth (10 g/l tryptone, 5 g/l yeast extract) with shaking at 37°C.

### Animal experiments

Six to eight week old female mice obtained from The Jackson Laboratory were used for this study.

Mice were injected intraperitoneally with 1×10^7^ colony forming units (CFU)/animal suspended in 0.1 ml phosphate buffered saline (PBS). Mice were euthanized and peritoneal lavages were collected an hour post injection by flushing the peritoneal cavity with 5 ml PBS.

To collect murine neutrophils for single-cell chemotaxis experiments, mice were euthanized and peripheral blood was collected by cardiac puncture. Neutrophils were then isolated using a neutrophil enrichment kit (Stemcell Technologies, Vancouver, Canada) using instructions provided by the manufacturer.

### Single-cell experiments

Neutrophils were suspended in chemotaxis medium (1.5×10^6^ cells/ml in Hank's Balanced Salt Solution with 10% autologous serum, unless mentioned otherwise). In some experiments, PBS (vehicle control) or mouse monoclonal anti-human C5a antibody (ab11876, Abcam) dissolved in PBS was added at a final concentration of 10 µg/ml. To generate bacterial micro-agglutinates, 10^9^ CFU/ml were suspended in PBS containing 0.02% sodium azide. Rabbit anti-Vi serum (for Ty2) or rabbit anti-O9 serum (for Δ*tviB-vexE* mutant) were added to the bacterial suspension at a 1∶1 ratio to generate micro-agglutinates. The micropipette setup has been described previously [29]. Briefly, micropipettes with inner diameter of 2–3 µm were mounted on a motorized 3-axis manipulator that was controlled by a custom-written software. Neutrophils and bacteria were dispensed into a microscopic chamber (Figure S1). Neutrophils and bacteria were lifted above the chamber bottom by aspiring them on a micropipette or by using optical tweezers. The micropipette-held bacterial micro-agglutinate or bacteria immobilized by optical tweezers were gradually brought towards the micropipette-held neutrophil and the chemotactic response was recorded. Each experiments was repeated with cells from at least 4 different donors.

### Boyden chamber assay

Human neutrophils were isolated from peripheral blood of healthy adult donors using Cytoselect Cell Migration Assay kit (Cellbiolabs Inc., San Diego, CA). Neutrophils were suspended in HBSS at the concentration of approximately 1.5×10^6^ cells/ml and 300 µl were loaded into the top chamber of a 24-well Boyden chamber plate (Cell Biolabs inc., San Diego, CA). Sterile PBS or a bacterial suspension (1.5×10^7^ CFU/ml) in chemotaxis medium was added to the bottom chamber. In some experiments, the complement inhibitor Futhan (6-amidino-2-naphthyl p-guanidinobenzoate dimethanesulfonate) [34] was added to the bottom chamber at a final concentration of 50 µg/ml. Human IL-8 (100 ng/ml, R&D Systems) or fMLP 10 nM (*N*-Formyl-L-methionyl-L-leucyl-L-phenylalanine, 10 nM) (Sigma, St. Louis, MO) were also added to the bottom chamber in some experiments. In some experiments, PBS (vehicle control) or mouse monoclonal anti-human C5a antibody (ab11876, Abcam) dissolved in PBS was added to the bottom chamber at a final concentration of 10 µg/ml. The plates were incubated for two hours at 37°C in tissue culture incubator. The cells that migrated to the bottom chamber were counted using a hemocytometer. Each experiments was repeated with cells from at least 3 different donors.

### Flow cytometry

The peritoneal lavage containing approximately 10^6^ cells per animal was suspended in 2 ml cold phosphate-buffered saline and stained with Aqua Live/Dead cell discriminator (Invitrogen catalog no. L34597) according to the manufacturer's instructions. Cells were then washed and resuspended in fluorescence-activated cell sorting (FACS) buffer (PBS containing 1% bovine serum albumin and 1 mM EDTA) and incubated for 15 min in blocking antibody anti-CD16/32 (eBioscience clone 93). Cells were then stained for 20 min in the dark at 4°C with optimized concentrations of anti-CD3 PE (eBioscience clone 17A2), anti-B220 PE (eBioscience clone RA3-6B2), anti-NK1.1 PE (eBioscience clone PK136), anti-Ly6C Pacific Blue (eBioscience clone HK1.4), anti-Ly6G PerCPCy5.5 (BD Pharmingen clone 1A8) and anti-CD11b APC Cy7 (Biolegend clone M1/70). Cells were washed twice with FACS buffer and subsequently fixed in 4% paraformaldehyde for 1 hour. Cells were then washed twice and resuspended in FACS buffer and analyzed using an LSR II flow cytometer (Becton Dickinson, San Jose, CA). GFP expressing bacteria were detected using 488 nm excitation. The data were analyzed by using FlowJo software (Treestar, Inc., Ashland, OR). Gates were set on singlets and then on live cells. Subsequent gates were based on Fluorescence-Minus-One and unstained controls. To confirm neutrophil morphology, LY6G^+^ LY6C^+^ cells were sorted using a MoFlo high-speed cell sorter (Dako). Cytospin samples were prepared from the sorted cells using a Shandon cytocentrifuge (Thermo Scientific, MI) and dried prior to staining with a Diff-Quik stain kit (IMEB, Inc., San Marcos, CA).

Vi capsule expression and C3b deposition was detected using flow cytometry as described previously [16], [55]. Bacterial cells were detected using the DNA specific stain propidium iodide. For detection of the Vi capsular polysaccharide, bacterial cells incubated in rabbit anti-Vi serum (Difco) were washed and stained with goat anti-rabbit FITC-conjugate (Jackson ImmunoLabs). To detect C3b deposition, bacterial cells were incubated for 30 minutes in 1% human serum. Cells were then washed and stained with FITC-conjugated goat anti-human C3b antibody (MP Biomedicals). For each sample, 50 000 events (bacterial cells) were collected using LSR II (Becton Dickinson). Data were expressed as percentage values of positive counts per total counts.

### Enzyme linked immune-sorbent assay (ELISA)

For the human serum ELISA assays, *S.* Typhi strains were grown statically overnight at 37°C in SOB+Mg medium (20 g/liter tryptone, 5 g/liter yeast extract, 10 mM NaCl, 10 mM KCl, 10 mM MgCl2, 10 mM MgSO4) to induce expression of the Vi capsular polysaccharide. Then 1×10^7^ CFU of S. Typhi were incubated for 15 minutes at 37°C in 10% human serum complement (Quidel) diluted in PBS. The generation of C5a was detected with the Human C5a ELISA Kit II (BD; cat #557965) using the instructions provided by the manufacturer.

### Statistical analysis

To determine statistical significance between treatment groups in the animal experiments an unpaired Student *t*-test was used. A *P* value of less than 0.05 was considered to be significant.

## Supporting Information

Figure S1 The alternative pathway of complement activation. The schematic illustrates the steps in the alternate pathway of complement activation that lead to the formation of C5a. Approaches used in this study to block this pathway at different steps in the cascade are indicated in red.
(PDF)

Figure S2 Single-cell approach to assess neutrophil chemotaxis toward bacteria in buffer containing serum. (A) A bacterial culture (image on the top left) and neutrophils from humans or mice (images on the top right) were deposited into a microscopy chamber with two open sides (schematic drawing in the center). Vertically adjustable water reservoirs allowed accurate pressure application to facing micropipettes inserted into this chamber. An initially quiescent neutrophil was aspirated at the tip of a micropipette. Bacteria were either immobilized with optical tweezers or micro-aggregates of bacteria were produced and picked up using a second micropipette. Bacteria (arrow) and neutrophils were then brought stepwise into close proximity (image at the bottom). (B) The image illustrates how the maximum extension of chemotactic pseudopodia was determined. The image shows a human neutrophil (bottom) extending a pseudopod towards *S.* Typhimurium (top). (C) Quantification of maximum pseudopod extension of human neutrophils towards the indicated bacterial strains.
(PDF)

Figure S3 The Vi capsular polysaccharide inhibits chemotactic responses of murine neutrophils. (A–B) The indicated bacterial strains were immobilized by laser tweezers and brought in close proximity to a pipette-held murine neutrophil. Video micrographs were taken at the indicated time points. Blood from 4 BALB/c mice was pooled for isolation of serum and neutrophils for an experiment. Each experiment was repeated at least four times and one representative example is shown.
(PDF)

Figure S4 Anti-C5a antibody inhibits neutrophil chemotaxis. Agglutinated cells of *S.* Typhimurium were pipette-held and brought in close proximity to a pipette-held human neutrophil in the absence (A) or the presence (B) of mouse anti-human C5a monoclonal antibody.
(PDF)

Figure S5 Quantification of neutrophil infiltrates elicited by infection with *S.* Typhi. Mice (BALB/c) were injected intraperitoneally with sterile PBS or the indicated GFP-labeled bacterial strains and cells were collected one hour later by intraperitoneal lavage. (A) Bacterial numbers recovered from peritoneal lavage. NS, not significantly different. (B) Quantitative analysis of neutrophil infiltration is shown as geometric means (bars) ± standard error from groups of six animals. (C) Representative images of neutrophil infiltration (LY6C^+^ LY6G^+^ cells) in intraperitoneal lavage populations detected by flow cytometry.
(PDF)

Figure S6 Gating strategy for detecting an association of *S.* Typhi with neutrophils from peritoneal lavage. Gating strategy for analyzing peritoneal lavage cell suspensions. After doublet elimination (top left panel) live cells were gated (top middle panel) and CD3^+^ B220^+^ NK1.1^+^ cells eliminated using a dump channel (top right panel). CD3^−^ B220^−^ NK1.1^−^ cells were then analyzed for expression of CD11B (right panel in the middle row). Next, CD3^−^ B220^−^ NK1.1^−^ CD11B^+^ cells were analyzed for expression of LY6C and LY6G cells (left panel in the middle row). CD3^−^ B220^−^ NK1.1^−^ CD11B^+^ LY6C^+^ LY6G^+^ cells (neutrophils) were analyzed by microscopy to confirm neutrophil morphology (bottom right panel). The gate for the detection of GFP-labeled *S.* Typhi in neutrophil populations was set using cells isolated from control mice injected with sterile PBS (bottom left and middle panels).
(PDF)

Video S1 *E. coli* cells were immobilized by laser tweezers and brought in close proximity to a pipette-held human neutrophil in the presence of human serum. At the conclusion of the experiment the neutrophil is released from the pipette by relieving the suction.
(MOV)

Video S2 *S.* Typhimurium cells were immobilized by laser tweezers and brought in close proximity to a pipette-held human neutrophil in the presence of human serum. At the conclusion of the experiment the neutrophil is released from the pipette by relieving the suction.
(MOV)

Video S3 *S.* Typhi cells were immobilized by laser tweezers and brought in close proximity to a pipette-held human neutrophil in the presence of human serum.
(MOV)

Video S4 *E. coli* cells were immobilized by laser tweezers and brought in close proximity to a pipette-held human neutrophil in the presence of heat-inactivated human serum. At the conclusion of the experiment the neutrophil is released from the pipette by relieving the suction.
(MOV)

Video S5 *S.* Typhimurium cells were immobilized by laser tweezers and brought in close proximity to a pipette-held human neutrophil in the presence of heat-inactivated human serum. At the conclusion of the experiment the neutrophil is released from the pipette by relieving the suction.
(MOV)

Video S6 Cells of the non-capsulated *S.* Typhi Δ*tviB-vexE* mutant were immobilized by laser tweezers and brought in close proximity to a pipette-held human neutrophil in the presence of human serum.
(MOV)

Video S7 *S.* Typhi cells were immobilized by laser tweezers and brought in close proximity to a pipette-held murine neutrophil in the presence of murine serum. At the conclusion of the experiment the neutrophil is released from the pipette by relieving the suction.
(MOV)

Video S8 Cells of the non-capsulated *S.* Typhi Δ*tviB-vexE* mutant were immobilized by laser tweezers and brought in close proximity to a pipette-held murine neutrophil in the presence of murine serum. At the conclusion of the experiment the neutrophil is released from the pipette by relieving the suction.
(MOV)

Video S9 Agglutinated cells of the non-capsulated *S.* Typhi Δ*tviB-vexE* mutant were pipette-held and brought in close proximity to a pipette-held murine neutrophil from C3-deficient mice in the presence of serum from C3-deficient mice.
(MOV)

Video S10 Agglutinated cells of the non-capsulated *S.* Typhi Δ*tviB-vexE* mutant were pipette-held and brought in close proximity to a pipette-held murine neutrophil from C3-deficient mice in the presence of serum from wild-type (C57BL/6) mice. At the conclusion of the experiment the neutrophil is released from the pipette by relieving the suction.
(MOV)

Video S11 Agglutinated cells of the non-capsulated *S.* Typhi Δ*tviB-vexE* mutant were pipette-held and brought in close proximity to a pipette-held murine neutrophil from wild-type (C57BL/6) mice in the presence of serum from wild-type (C57BL/6) mice.
(MOV)

Video S12 Agglutinated cells of the non-capsulated *S.* Typhi Δ*tviB-vexE* mutant were pipette-held and brought in close proximity to a pipette-held murine neutrophil from wild-type (BALB/c) mice in the presence of murine serum.
(MOV)

Video S13 Agglutinated cells of the non-capsulated *S.* Typhi Δ*tviB-vexE* mutant were pipette-held and brought in close proximity to a pipette-held murine neutrophil from C5aR-deficient mice in the presence of murine serum.
(MOV)
