# Supplementary figures and images for: The Vi Capsular Polysaccharide Enables Salmonella enterica Serovar Typhi to Evade Microbe-Guided Neutrophil Chemotaxis
Source: PLoS Pathog. 2014 Aug 7;10(8):e1004306. doi: 10.1371/journal.ppat.1004306 (PMC4125291; doi:10.1371/journal.ppat.1004306)

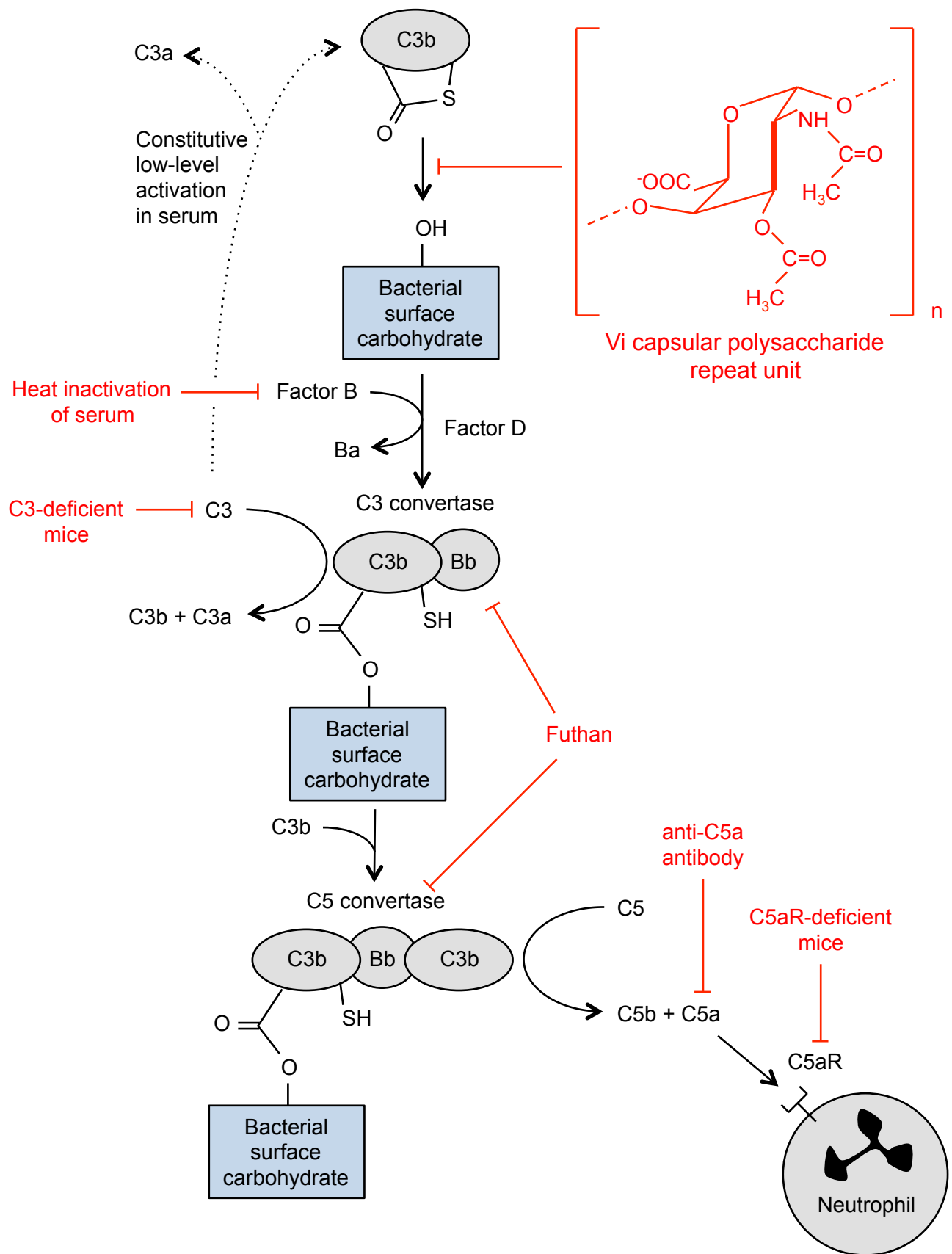

Supplement: Figure S1 — The alternative pathway of complement activation. The schematic illustrates the steps in the alternate pathway of complement activation that lead to the formation of C5a. Approaches used in this study to block this pathway at different steps in the cascade are indicated in red. (PDF) [file ppat.1004306.s001.pdf]

A

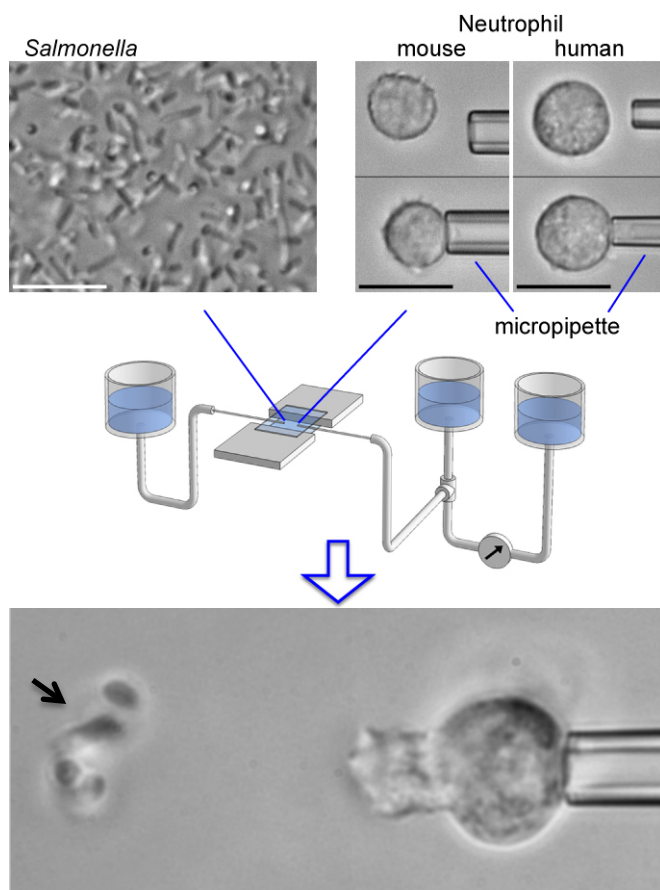

B

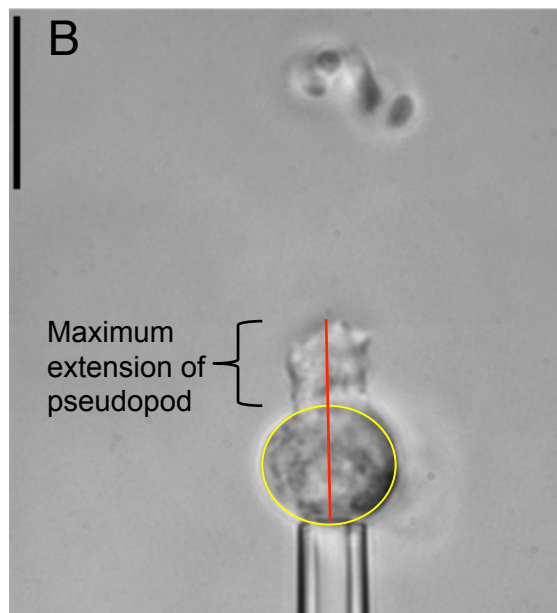

C

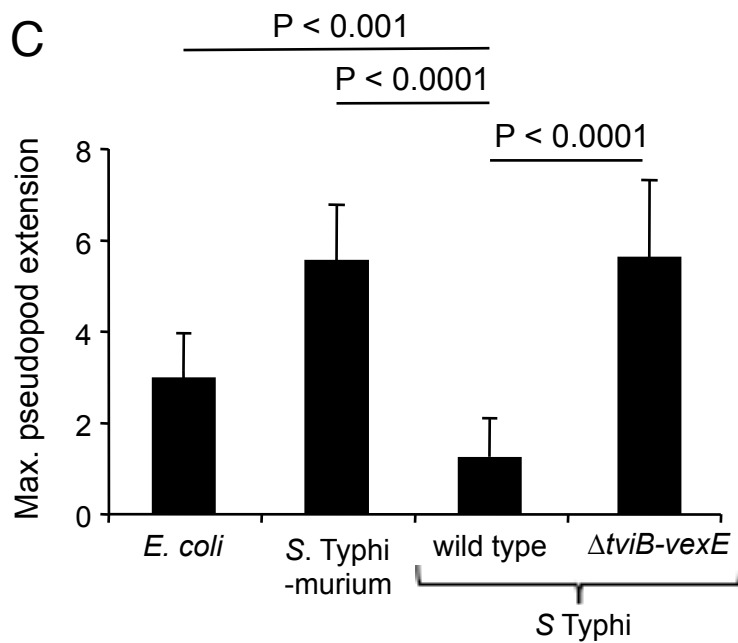

Supplement: Figure S2 — Single-cell approach to assess neutrophil chemotaxis toward bacteria in buffer containing serum. (A) A bacterial culture (image on the top left) and neutrophils from humans or mice (images on the top right) were deposited into a microscopy chamber with two open sides (schematic drawing in the center). Vertically adjustable water reservoirs allowed accurate pressure application to facing micropipettes inserted into this chamber. An initially quiescent neutrophil was aspirated at the tip of a micropipette. Bacteria were either immobilized with optical tweezers or micro-aggregates of bacteria were produced and picked up using a second micropipette. Bacteria (arrow) and neutrophils were then brought stepwise into close proximity (image at the bottom). (B) The image illustrates how the maximum extension of chemotactic pseudopodia was determined. The image shows a human neutrophil (bottom) extending a pseudopod towards S. Typhimurium (top). (C) Quantification of maximum pseudopod extension of human neutrophils towards the indicated bacterial strains. (PDF) [file ppat.1004306.s002.pdf]

S. Typhi wild type

S. Typhi  $\Delta tviB$ -*vexE* mutant

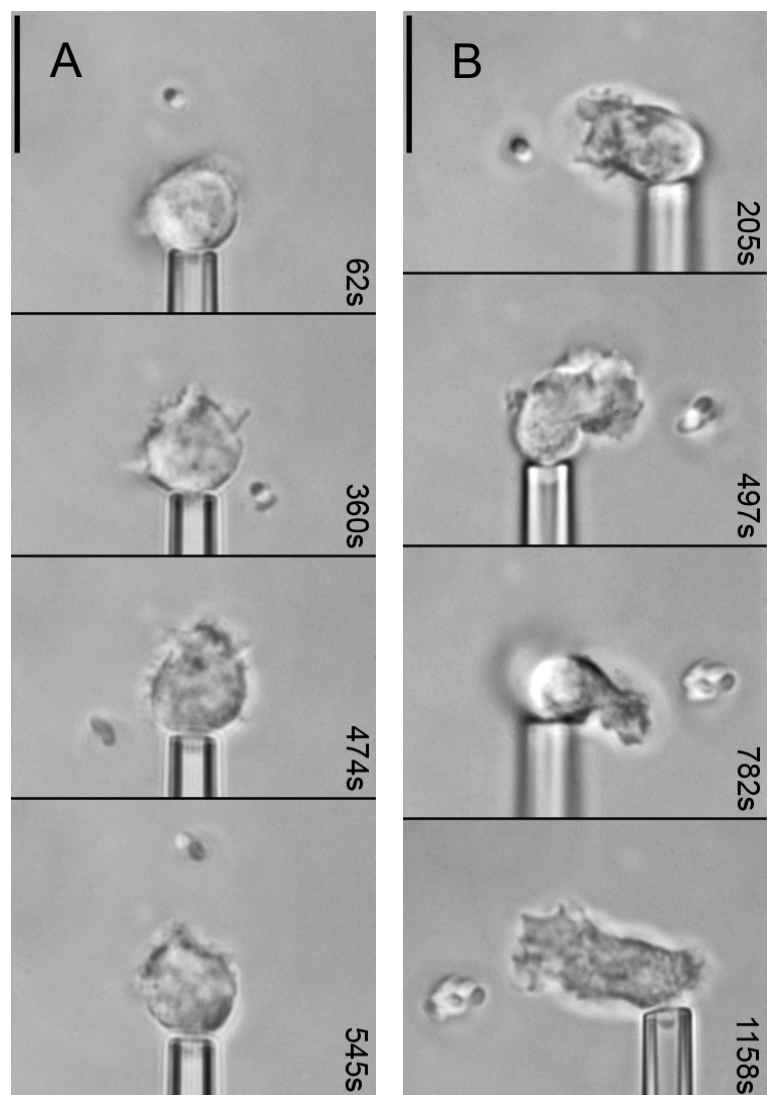

Supplement: Figure S3 — The Vi capsular polysaccharide inhibits chemotactic responses of murine neutrophils. (A–B) The indicated bacterial strains were immobilized by laser tweezers and brought in close proximity to a pipette-held murine neutrophil. Video micrographs were taken at the indicated time points. Blood from 4 BALB/c mice was pooled for isolation of serum and neutrophils for an experiment. Each experiment was repeated at least four times and one representative example is shown. (PDF) [file ppat.1004306.s003.pdf]

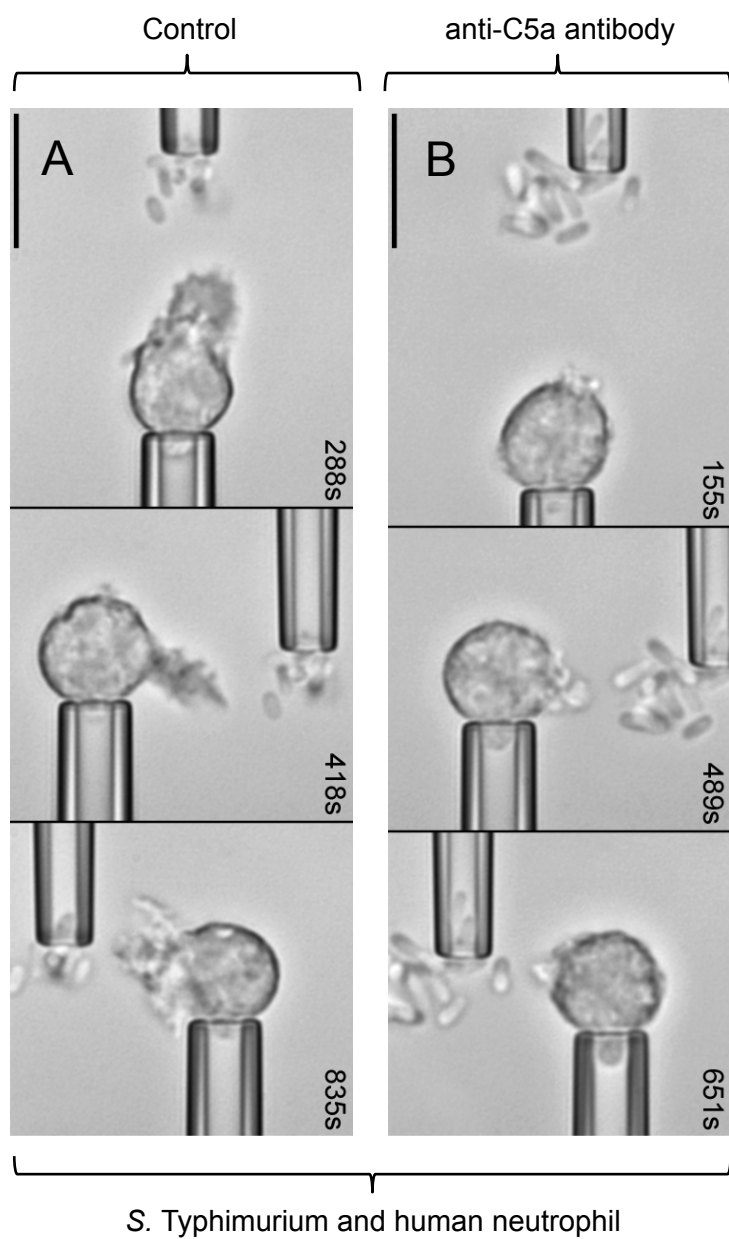

Supplement: Figure S4 — Anti-C5a antibody inhibits neutrophil chemotaxis. Agglutinated cells of S. Typhimurium were pipette-held and brought in close proximity to a pipette-held human neutrophil in the absence (A) or the presence (B) of mouse anti-human C5a monoclonal antibody. (PDF) [file ppat.1004306.s004.pdf]

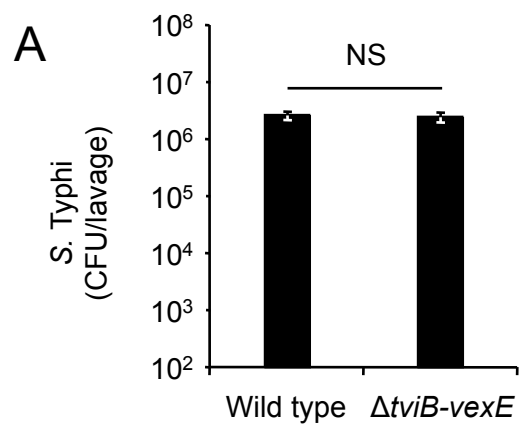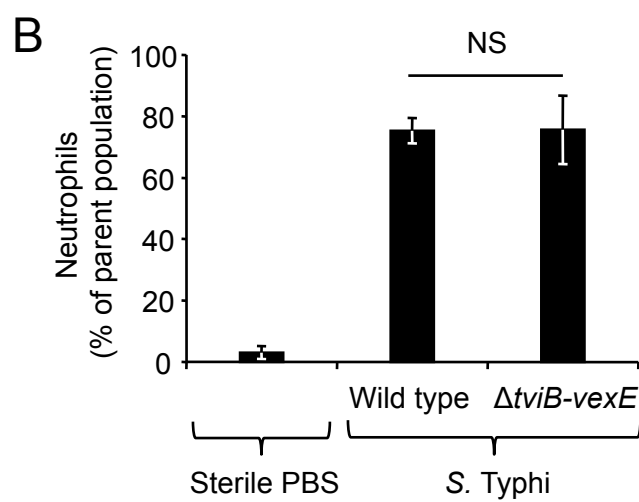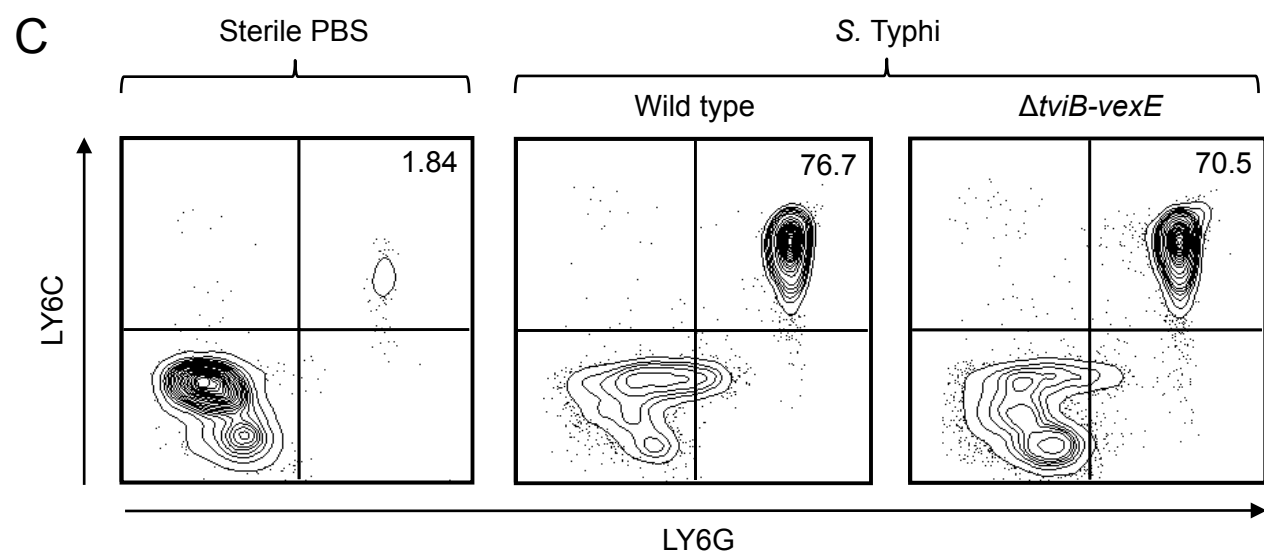

Supplement: Figure S5 — Quantification of neutrophil infiltrates elicited by infection with S. Typhi. Mice (BALB/c) were injected intraperitoneally with sterile PBS or the indicated GFP-labeled bacterial strains and cells were collected one hour later by intraperitoneal lavage. (A) Bacterial numbers recovered from peritoneal lavage. NS, not significantly different. (B) Quantitative analysis of neutrophil infiltration is shown as geometric means (bars) ± standard error from groups of six animals. (C) Representative images of neutrophil infiltration (LY6C+ LY6G+ cells) in intraperitoneal lavage populations detected by flow cytometry. (PDF) [file ppat.1004306.s005.pdf]

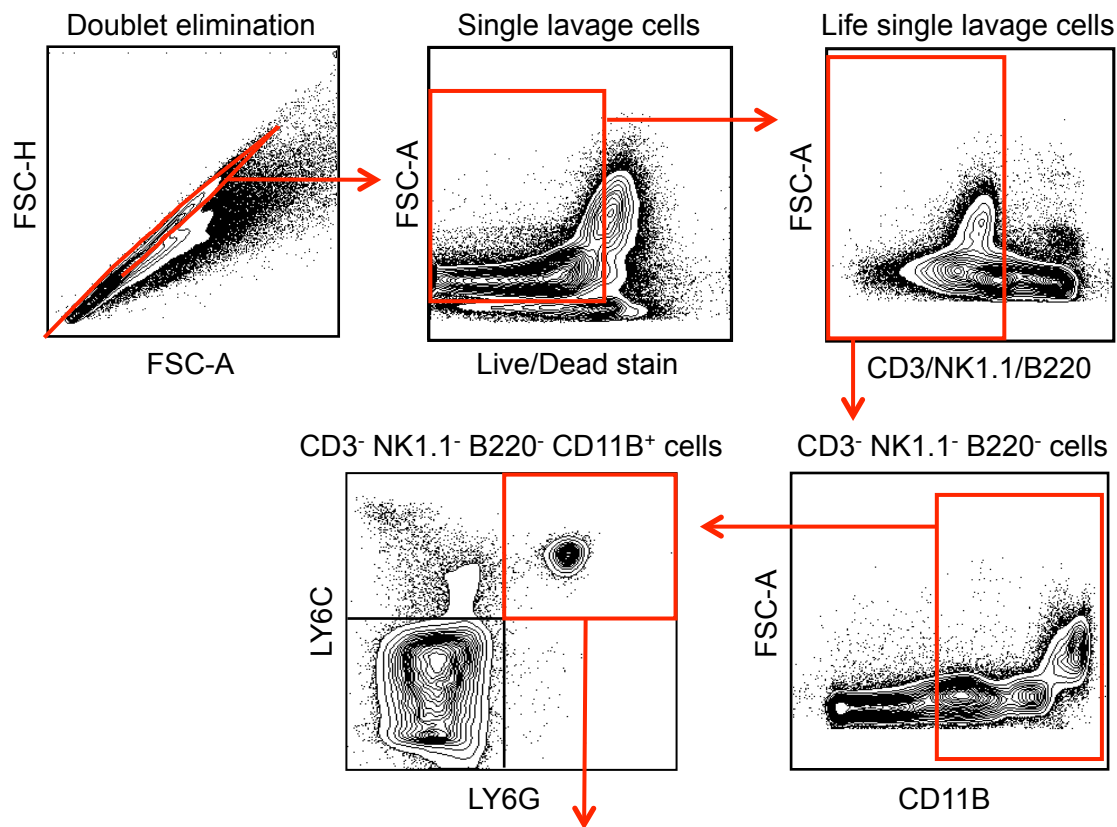

CD3<sup>-</sup> NK1.1<sup>-</sup> B220<sup>-</sup> CD11B<sup>+</sup> LY6C<sup>+</sup> LY6G<sup>+</sup> cells (neutrophils)

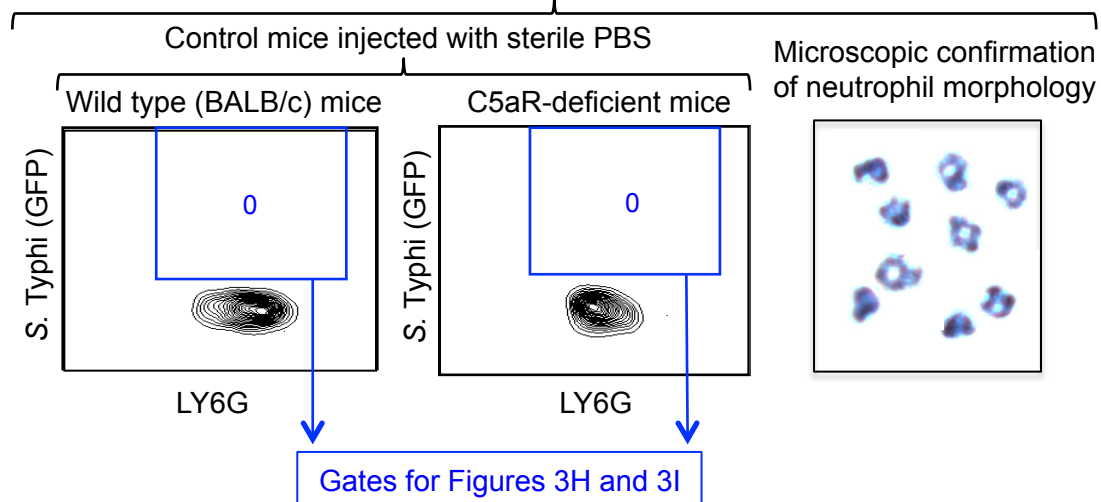

Supplement: Figure S6 — Gating strategy for detecting an association of S. Typhi with neutrophils from peritoneal lavage. Gating strategy for analyzing peritoneal lavage cell suspensions. After doublet elimination (top left panel) live cells were gated (top middle panel) and CD3+ B220+ NK1.1+ cells eliminated using a dump channel (top right panel). CD3− B220− NK1.1− cells were then analyzed for expression of CD11B (right panel in the middle row). Next, CD3− B220− NK1.1− CD11B+ cells were analyzed for expression of LY6C and LY6G cells (left panel in the middle row). CD3− B220− NK1.1− CD11B+ LY6C+ LY6G+ cells (neutrophils) were analyzed by microscopy to confirm neutrophil morphology (bottom right panel). The gate for the detection of GFP-labeled S. Typhi in neutrophil populations was set using cells isolated from control mice injected with sterile PBS (bottom left and middle panels). (PDF) [file ppat.1004306.s006.pdf]
